# Microbial communities in the liver and brain are informative for postmortem submersion interval estimation in the late phase of decomposition: A study in mouse cadavers recovered from freshwater

**DOI:** 10.3389/fmicb.2022.1052808

**Published:** 2022-11-15

**Authors:** Linlin Wang, Fuyuan Zhang, Kuo Zeng, Wenwen Dong, Huiya Yuan, Ziwei Wang, Jin Liu, Jiaqing Pan, Rui Zhao, Dawei Guan

**Affiliations:** ^1^Department of Forensic Pathology, China Medical University School of Forensic Medicine, Shenyang, China; ^2^Liaoning Province Key Laboratory of Forensic Bio-evidence Science, Shenyang, China; ^3^Institute of Evidence Law and Forensic Science, China University of Political Science and Law, Beijing, China

**Keywords:** aquatic habitat, decomposition, internal organ, microbial community, postmortem submersion interval

## Abstract

**Introduction:**

Bodies recovered from water, especially in the late phase of decomposition, pose difficulties to the investigating authorities. Various methods have been proposed for postmortem submersion interval (PMSI) estimation and drowning identification, but some limitations remain. Many recent studies have proved the value of microbiota succession in viscera for postmortem interval estimation. Nevertheless, the visceral microbiota succession and its application for PMSI estimation and drowning identification require further investigation.

**Methods:**

In the current study, mouse drowning and CO_2_ asphyxia models were developed, and cadavers were immersed in freshwater for 0 to 14 days. Microbial communities in the liver and brain were characterized *via* 16S rDNA high-throughput sequencing.

**Results:**

Only livers and brains collected from 5 to 14 days postmortem were qualified for sequencing. There was significant variation between microbiota from liver and brain. Differences in microbiota between the cadavers of mice that had drowned and those only subjected to postmortem submersion decreased over the PMSI. Significant successions in microbial communities were observed among the different subgroups within the late phase of the PMSI in livers and brains. Eighteen taxa in the liver which were mainly related to *Clostridium_sensu_stricto* and *Aeromonas*, and 26 taxa in the brain which were mainly belonged to *Clostridium_sensu_stricto*, *Acetobacteroides*, and *Limnochorda*, were selected as potential biomarkers for PMSI estimation based on a random forest algorithm. The PMSI estimation models established yielded accurate prediction results with mean absolute errors ± the standard error of 1.282 ± 0.189 d for the liver and 0.989 ± 0.237 d for the brain.

**Conclusions:**

The present study provides novel information on visceral postmortem microbiota succession in corpses submerged in freshwater which sheds new light on PMSI estimation based on the liver and brain in forensic practice.

## Introduction

Human cadavers are often discovered in a range of natural aquatic habitats such as lakes, rivers, and oceans due to drowning, disasters, and accidents (Cartozzo et al., 2021b). Bodies retrieved from water pose difficulties to the investigating authorities, particularly corpses at an advanced stage of decay. A forensic pathologist is generally required to determine the cause of death and the postmortem submersion interval (PMSI; Humphreys et al., 2013). Many studies have been conducted to address these questions. Though accumulated degree-days based on the morphological state of decomposition has been suggested to determine the PMSI (Heaton et al., 2010), it is unsuitable for corpses that have been submerged in very cold water (Dickson et al., 2011; Palazzo et al., 2020). In addition, typical macroscopic signs including the classic plume of white froth from the nose or mouth, overinflated, crepitant lungs, pulmonary edema, and water in the stomach have frequently been used to identify drowning as a cause of death, but such indicators gradually become less reliable with the progression of decomposition (Schneppe et al., 2021). Given this, novel methods for PMSI estimation and the identification of drowning are required for use in forensic practice.

Aquatic bacteria have recently attracted widespread interest from forensic experts (Uchiyama et al., 2012; Lang et al., 2016). Bacteria are ubiquitous in natural bodies of water, and they are small (0.2–2.0 μm), which facilitates their entrance into blood circulation and their deposition in the viscera during drowning (Oliveira and Amorim, 2018). A previous study indicates that various bacteria spread around the entire corpse after death (Wójcik et al., 2021). Specific genera, including *Aeromonas* in freshwater and *Vibrio* and *Photobacterium* in seawater, are indicators of drowning when they are detected in the blood and viscera of victims *via* culture-dependent and/or PCR-based methods (Kakizaki et al., 2008; Aoyagi et al., 2009). Ubiquitous microbes including the internal microbiota of the carcass as well as those of the surrounding environment play an important role in the natural decomposition of carcasses in aquatic systems (Metcalf et al., 2016). However, these methods (i.e., culture-dependent and/or PCR-based methods) could provide only limited information. With the advancement of sequencing technologies, especially next-generation sequencing, it is possible to obtain a more comprehensive understanding of microbial community succession during the decay process. Many studies in human and animal corpses indicate the potential value of microbial succession for PMI or PMSI estimation (Li et al., 2021; Randall et al., 2021). To date, microbial studies investigating aquatic ecosystems in this context have mainly focused on microbes that have colonized the surface of remains or specific body parts (e.g., bones; Wallace et al., 2021; Cartozzo et al., 2021a), which are vulnerable to environmental changes (Kaszubinski et al., 2022). However, there is a lack of studies assessing the succession pattern of microbial communities colonized in the internal organs, which are relatively resistant to environmental abiotic factors (i.e., pH and temperature) and biotic factors (i.e., insects and scavenger activities; Tomberlin et al., 2011). The liver and brain are believed to be sterile in living hosts (Javan et al., 2016). The microbes discovered in these organs of cadavers could represent those directly associated with decomposition, making them ideal subjects for postmortem microbiota investigation.

Using 16S rDNA sequencing, our previous study demonstrated that microbial communities in the viscera differed in drowning and postmortem submersion groups at 3 days postmortem (Wang et al., 2020). In another study microbiota succession in the gut was helpful for estimating the PMSI (Zhang et al., 2022b). Whether microbial succession in other visceral organs could be used for PMSI estimation and the determination of cause of death requires investigation. In the present study, to verify this hypothesis, mouse drowning and postmortem submersion models were developed, and corpses were maintained in freshwater for 0 to 14 days. Microbial communities in liver and brain were characterized by 16S rDNA high-throughput sequencing, and data were analyzed with machine-learning algorithms.

## Materials and methods

### Sample collection and experimental setup

All animal experiments were approved by the Animal Experiment Committee of China Medical University (approval number CMU2021202). All experiments were performed in October in a natural freshwater river (Shenyang, China; N41°57′, E123°27′). Adult male C57BL/6 J mice (20–25 g, aged 8–10 weeks, *n* = 180) were purchased from the Experimental Animal Center of China Medical University, then housed in micro-isolator cages under standard lighting (light/dark periods of 12 h) with free access to drinking water and food. Five water samples (1 l each) were taken from the experimental sites before the animal experiments and filtered through sterile 0.2-μm filters (Fisher Scientific, Hampton, NH). A total of 144 mice were randomly distributed into drowning (*n* = 72) and postmortem submersion (*n* = 72) groups. The drowning model was established as previously reported (Zhang et al., 2022a). Briefly, mice were deposited in sterile string bags and immersed in 30-cm-deep water for 1 min before being retrieved from the water for 30 s. The above steps were repeated until the animals died, then the corpses were submerged underwater. Mice in the postmortem submersion group were killed by CO_2_ inhalation then submersed underwater. Nine timepoints were investigated; immediately after death, 6 h and 12 h after death, and 1, 3, 5, 7, 10, and 14 days after death. At each timepoint liver (the right lobe) and brain (the right hemispheres) specimens were harvested from 16 mice (8 per group). To assess the percentages of intestine-derived bacteria in liver and brain microbial communities during decomposition, 16 cecal content samples were collected from the corpses immediately after death. All samples were immediately frozen in liquid nitrogen and stored at −80°C for subsequent sequencing. The remaining 36 mice were processed in accordance with the above-described procedures (drowning group 18 mice, postmortem submersion subgroup 18 mice; 2 mice at each indicated timepoint) as an independent validation experiment. The specific grouping is presented in Supplementary Table 1.

### 16S rDNA extraction and amplification

Bacterial genomic DNA from all samples, including cecal content (*n* = 16), liver (*n* = 180), brain (*n* = 180), and water (*n* = 5) was extracted using the CTAB method. DNA concentration and purity were then determined *via* 1% agarose gels. The V3-V4 region of 16S rDNA was amplified by PCR (98°C for 1 min, followed by 30 cycles of 98°C for 10 s, 50°C for 30 s, and 72°C for 30 s, then final extension at 72°C for 10 min) using the primers 341F (CCTACGGGNGGCWGCAG) and 806R (GGACTACHVGGGTATCTAAT), which were synthesized by Sangon Biotech (Sangon, Shanghai, China). DNA was then sequenced on the Illumina NovaSeq platform (Illumina, United States), and 250-bp paired-end reads were generated.

### Sequence analysis

The 16S rRNA gene sequences were processed using QIIME 1.9.1 (Caporaso et al., 2010), USEARCH 10.0 (Edgar, 2010), and in-house scripts. Paired-end Illumina reads were checked by FastQC (de Sena Brandine and Smith, 2019), and further processed by USEARCH (including joining of paired-end reads, relabeling of sequencing names, removal of barcodes and primers, filtering of low-quality reads, and finding non-redundancy reads). Based on high-confidence 16S representative sequences, an amplicon sequence variants (ASVs) table was generated. The taxonomy of the representative sequences was classified with the “RDP trainset 16” database (Cole et al., 2014) on the basis of the sintax algorithm in USEARCH (−sintax command). ASVs assigned to chloroplasts and mitochondria were removed. An ASV table was generated within USEARCH (−otutab command). For alpha and beta diversity, samples were first rarefied at minimal sequences by USEARCH (−otutab_norm command).

### Analysis of microbial communities

Data analyses were conducted using R (v.4.1.1).^1^ Alpha diversity was measured by the Chao1 and Shannon indexes with the “vegan” package^2^ in R. The Chao1 index was used to estimate alpha diversity richness and the Simpson index to evaluate evenness in addition to richness. Analysis of the difference in alpha diversity between drowning and postmortem submersion groups was performed using Wilcoxon rank-sum tests, and corresponding *p* values were corrected for multiple tests using a false discovery rate set at 0.05. Differences in beta diversity metrics (unweighted UniFrac and Bray–Curtis) were assessed visually using principal coordinates analysis (PCoA) and statistically using permutational multivariate analysis of variance tests (PERMANOVAs), with a total of 999 permutations (“vegan” package). Unweighted UniFrac considers phylogeny and taxa, while Bray–Curtis takes taxa and relative abundances into account. Multiple PERMANOVAs were performed, and the groups tested included cause of death (drowning and postmortem submersion), sample type (liver and brain), and PMSI (5, 7, 10, and 14 days). Fast expectation–maximization microbial source tracking (FEAST) was used to calculate the contributions of water and intestine bacterial communities as described previously (Shenhav et al., 2019), with the “FEAST” package of R. FEAST can identify the origins of complex microbial communities based on a statistical model that assumes each sink is a complex combination of known and unknown sources. In this study, water and intestine samples were defined as “sources,” and liver and brain samples were defined as “sinks.”

### Random forest models

Datasets derived from microbiomics have the characteristics of high dimensionality and large amounts of noise and redundancy (Li et al., 2020; Vidanaarachchi et al., 2020), and they are not amenable to analysis with traditional analytical methods (Zhang et al., 2019; Park et al., 2021). Random forest (RF) has become a popular tool for the analysis of microbial data, given that it is relatively robust with respect to outliers and noise, and is not prone to over-fitting (Knights et al., 2011). RF reportedly exhibits satisfactory performance when used to analyze microbial data to address unanswered forensic questions such as cause of death and postmortem interval (Metcalf et al., 2013; Zhang et al., 2019).

The present study investigated the use of different organs for PMSI estimation and drowning determination. RF regression and classification models were established based on microbiota profiles (the abundance data of each ASV) using default parameters of the R implementation of the algorithm (R package “randomForest”; ntree = 1,000, square root of the number of variables for the classification model and one-third of the variables for the regression model). To visualize the similarity of samples from different groups, a multidimensional scaling (MDS) ordination plot was generated using the MDSplot function of the “randomForest” package. Final performance was assessed *via* the mean absolute error (MAE) for the regression model, and the area under the receiver operating characteristic (ROC) curve for classification. Bacterial ASVs were ranked in order of their feature importance (the percentage increase in the mean-squared error; %IncMSE) in the regression model. Biomarker sets were generated by selecting the minimum error using 10-fold cross-validation.

## Results

### Overview of liver and brain microbial communities during decomposition

A total of 360 viscera samples including 180 from the liver and 180 from the brain were collected and analyzed in the exploratory and validation experiments at nine PMSIs spanning 14 days (Supplementary Table 1). Macroscopically, no significant signs of decomposition were observed within 5-day postmortem. Liver and brain showed minimal autolysis at 7 days. Mild liquefaction was observed in liver and brain at 10 days. At 14 days, there were scattered putrefactive blisters on the surface and parenchyma of the liver. The brain presented apparent liquefaction. After PCR amplification and agarose gel electrophoresis detection, almost all samples collected before 5-day postmortem were not qualified enough for use in subsequent experiments (The target region of 16S rDNA could not be amplified efficiently after multiple PCR, implying the low abundance of bacteria; Supplementary Table 1). Accordingly, only samples with PMSIs ranging from 5 to 14 days were further analyzed. The V3-V4 hypervariable region of the 16S rDNA gene was sequenced to characterize the microbial community. Following quality filtering and rarefaction, a total of 7,089,046 high-quality sequences were generated from 181 sample libraries, which were clustered into 3,071 ASVs. Rarefaction curves indicated that as sequence depth increased, species richness rose considerably and then reached asymptotes (Supplementary Figure 1), demonstrating that the tissues were sufficiently sequenced to observe all taxa.

**Figure 1:**
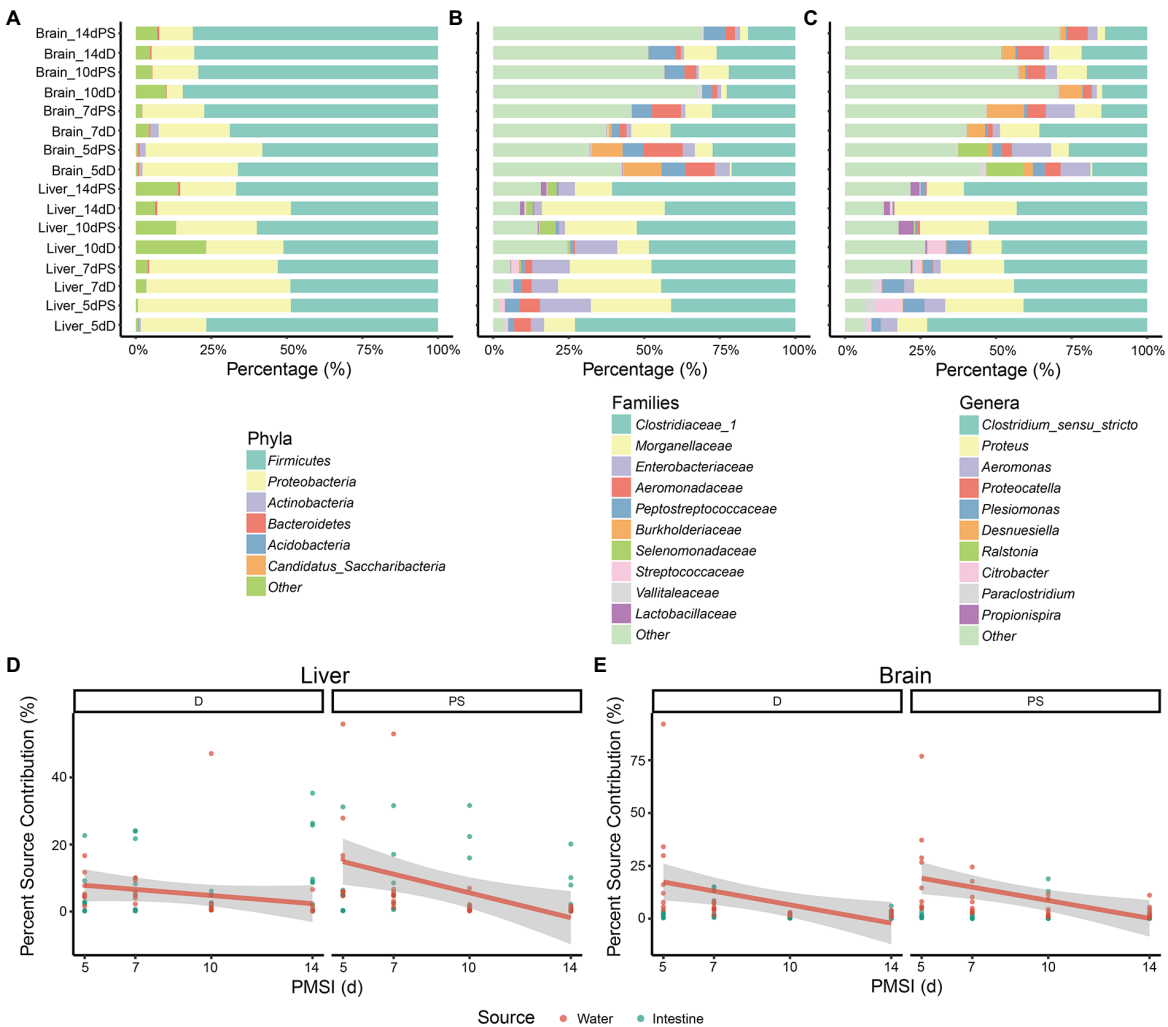
Composition of microbial communities in internal organs and microbial source tracking. **(A–C)** Relative abundance of bacterial taxa at different taxonomic levels. Stacked bar charts of the top 6 bacterial phyla **(A)**, top 10 bacterial families **(B)**, and top 10 bacterial genera **(C)** with the largest mean relative abundance in the liver and brain. Percentage source contributions of water-derived and intestine-derived bacteria to microbial communities in liver **(D)**, and brain **(E)**, over time were determined using FEAST. D, drowning group; PS, postmortem submersion group.

After taxonomy classification, composition analysis of microbial communities in the liver and brain was performed at different levels. At the phylum level, *Firmicutes* and *Proteobacteria* were dominant in all samples (Figure 1A). The relative abundance of *Proteobacteria* was higher in liver samples than in brain samples. The opposite was true for *Firmicutes*. In brain samples, the abundance of *Firmicutes* was increased and reached a plateau at 10 days, in conjunction with a decrease in *Proteobacteria*. As the taxonomy level increased, the difference between liver and brain became greater. At the family level higher abundance of *Clostridiaceae 1*, *Morganellaceae*, and *Enterobacteriaceae* was observed in liver samples compared to brain samples during the decomposition process, whereas the abundance of *Peptostreptococcaceae* was lower (Figure 1B). The relative abundance of *Aeromonadaceae* declined from 5 days and became relatively stable after 10 days both in liver samples and in brain samples. At the genus level, *Clostridium_sensu_stricto* and *Proteus* were more prevalent in liver samples, whereas *Proteocatella* and *Desnuesiella* were more common in brain samples (Figure 1C). The abundances of *Aeromonas* at 10 days and 14 days were lower than those at 5 days and 7 days in both organs.

To assess the possible source of microbes acquired from liver and brain samples during decomposition, an additional 16 cecal content samples and 5 water samples were obtained. The compositions of microbial communities in the liver and brain were compared with those of the water and intestine. The microorganism compositions in different samples were distinctly different (Figure 1; Supplementary Figure 2). None of the top 10 genera in the water, gut, and viscera were the same, indicating that great care must be taken to avoid microbial contamination from water or other organs during sampling in forensic practice. Given that water-derived bacteria may penetrate the viscera *via* the circulation during drowning, and the bacteria in the intestinal tract could disseminate to different parts of the body during decomposition, FEAST analysis was performed to assess the effects of water-derived and intestine-derived bacteria on viscera microbiota succession. For the liver samples, the contribution of water-derived bacteria decreased in the drowning and postmortem submersion groups as PMSI increased (Figure 1D). Similar results were observed in brain samples (Figure 1E). The contribution of intestine-derived bacteria to the brain microbial community was close to zero throughout, which was lower than that in liver (mean 7.8% ± 1.2%).

### Microbial diversity in liver and brain samples

**Figure 2:**
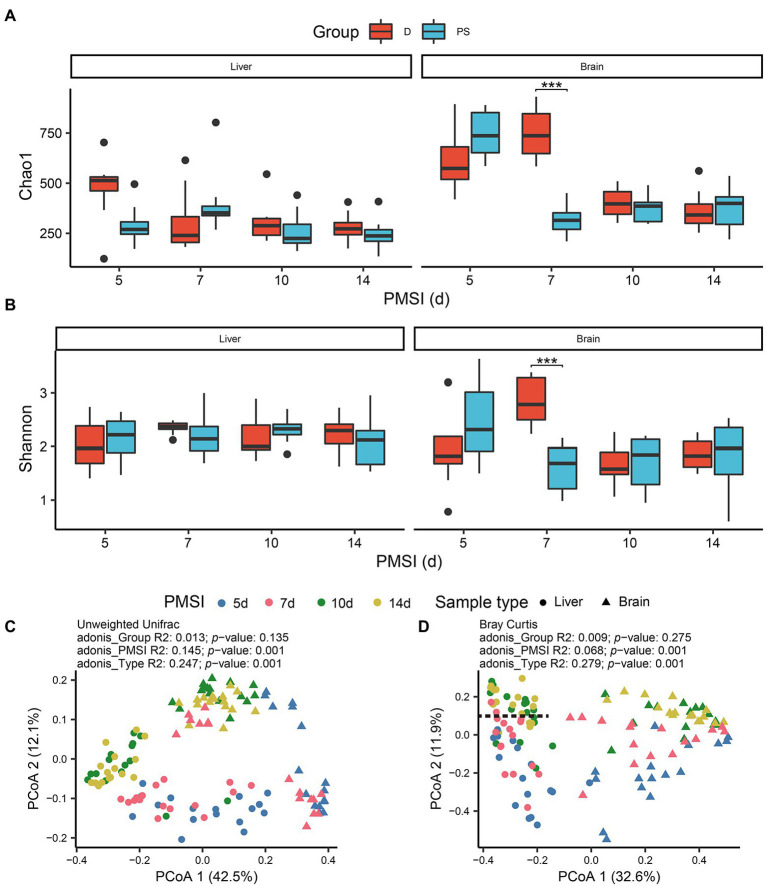
Alpha and beta diversities of microbiota in the liver and brain. Comparisons of the Chao1 **(A)**, and Shannon **(B)**, indices between drowning and postmortem groups at each PMSI. Ordination plot for the first two PCoA axes based on unweighted Unifrac **(C)**, and Bray–Curtis **(D)**, distances. Different colors indicate different PMSIs. Different sample types (liver or brain) are represented by different shapes. D, drowning group; PS, postmortem submersion group.

Alpha diversity was estimated using the Chao1 and Shannon indices (Figures 2A,B; Table 1). For the liver samples, there was no significant difference in the Chao1 and Shannon indexes between drowning and postmortem submersion groups at each timepoint. For the brain samples, there were only marked differences at 7-day postmortem. To visualize similarities and dissimilarities in postmortem bacterial compositions in different samples, PCoA was performed and represented in two-dimensional space. In an unweighted UniFrac distance-based PCoA plot (Figure 2C), principal coordinate 1 (PCo1) and PCo2 (42.5 and 12.1% of variance explained, respectively) axes showed that the microbial communities in the liver and brain were clearly separated during 14 days of decomposition (PERMANOVA, *R*^2^ = 0.247, *p* = 0.001). PCoA2 separated the communities mainly by PMSI (5–7 days and 10–14 days). Separation between sample types and among PMSIs was more notable in a Bray–Curtis-based PCoA plot (Figure 2D). However, no difference between drowning and postmortem submersion groups was observed based on unweighted UniFrac (*p* = 0.135) or Bray–Curtis distance (*p* = 0.275) analysis (Figures 2C,D). The PERMANOVA test indicated that both sample type and PMSI could significantly affect the microbial community (*p* < 0.05). Sample type explained more variance (*R*^2^ = 0.247 or 0.270) in microbial community compared to PMSI (*R*^2^ = 0.145 or 0.068) and cause of death (*R*^2^ = 0.013 or 0.009; Figures 2C,D). These results indicated that there were significant differences in microbial communities between the two types of viscera and among PMSIs.

**Table 1:** Comparisons of alpha diversity indexes (Chao1 and Shannon) between drowning and postmortem submersion by Wilcoxon rank-sum test.

PMSI	Sample type	*p*_Chao1	*p*.adjust_Chao1*	*p*_Shannon	*p*.adjust_Shannon*
5d	Liver	0.028	0.112	0.574	0.574
7d	Liver	0.083	0.166	0.195	0.574
10d	Liver	0.234	0.312	0.328	0.574
14d	Liver	0.442	0.442	0.442	0.574
5d	Brain	0.083	0.166	0.328	0.656
7d	Brain	0	0.001	0	0.001
10d	Brain	0.505	0.673	0.878	0.878
14d	Brain	0.721	0.721	0.721	0.878

* *p*-values were adjusted using Benjamini-Hochberg (BH) correction and the adjusted p value cut-off was 0.05.

### Applicability of liver and brain microbial communities for drowning determination

**Figure 3:**
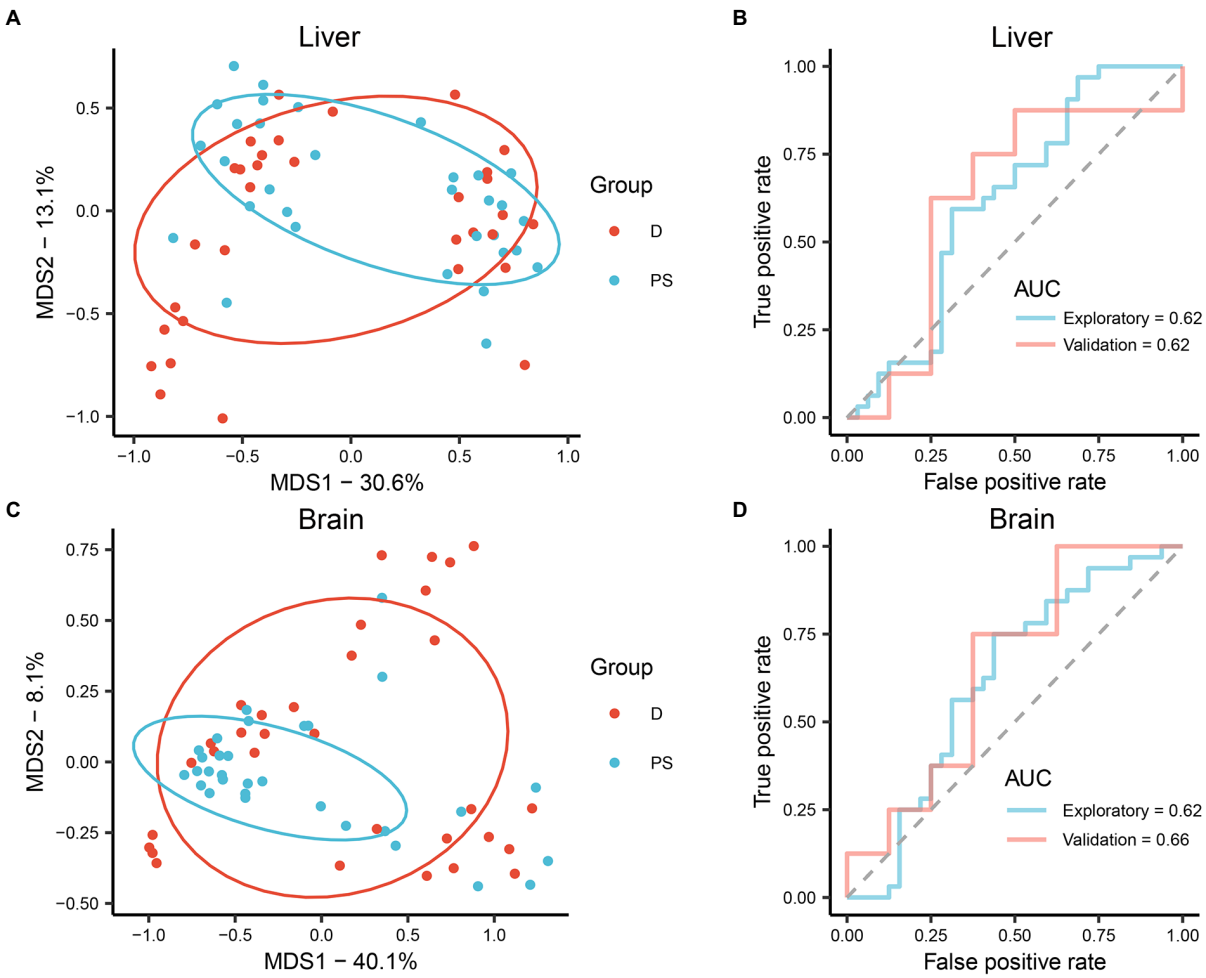
**(A,B)** Performance of the RF classification model built on microbiota in the liver. **(C,D)** Performance of the RF classification model built on microbiota in the brain. **(A,C)** MDS plot generated by the learning algorithm RF comparing the microbial community between drowning and postmortem submersion groups. **(B,D)** ROC curves of the RF classification model on data from exploratory and validation experiments. D, drowning group; PS, postmortem submersion group.

To further assess the applicability of microbiota in different organs for drowning determination, cause-of-death classification models were established based on the relative abundance of microbiota at the level of ASV using the RF machine-learning algorithm. In MDS plots, drowning and postmortem submersion groups were indistinguishable both in liver and brain models (Figures 3A,C). Similar results were observed in ROC curves. The areas under the curve (AUCs) were low (liver AUC exploratory 0.62, AUC validation 0.62; brain AUC exploratory 0.62, AUC validation 0.66; Figures 3B,D), indicating the performance of the classification models was poor. Thus, there was no significant difference in the microbial communities of liver and brain between the drowning and postmortem submersion groups when the individuals at different PMSIs were taken as a whole. Considering that the effect of PMSI on the bacterial community (*R*^2^ = 0.145 or 0.068) was stronger than that of cause of death (*R*^2^ = 0.013 or 0.009), we further analyzed the difference in microbiota between the groups at each timepoint. In PCoA analysis microbiomes from drowned corpses were clearly separated from those of postmortem submersion corpses at 5 days and 7 days, both in liver and brain (Supplementary Figures 3, 4). The same patterns were reflected in the MDS plots and ROC curves from RF classification models at each PMSI (Supplementary Figures 5, 6). Classification models were then generated based on the microbial communities in the brain and liver, which were collected at 5 days and 7 days. Overall performance is shown in Supplementary Figures 7 and 8 (liver AUC exploratory 0.94, AUC validation 0.56; brain AUC exploratory 0.79, AUC validation 0.94). These results demonstrated that the difference in microbial communities between drowned corpses and postmortem submersion corpses reduced gradually over the PMSI, and may only be helpful for drowning diagnosis for corpse retrieved at 5-day and 7-day postmortem. Overall, bacterial communities in liver and brain from corpses retrieved later could not be utilized for drowning determination. Thereafter, data from the two groups were assessed together to trace the common community succession for PMSI estimation.

### PMSI estimation based on microbial community succession in liver and brain

**Figure 4:**
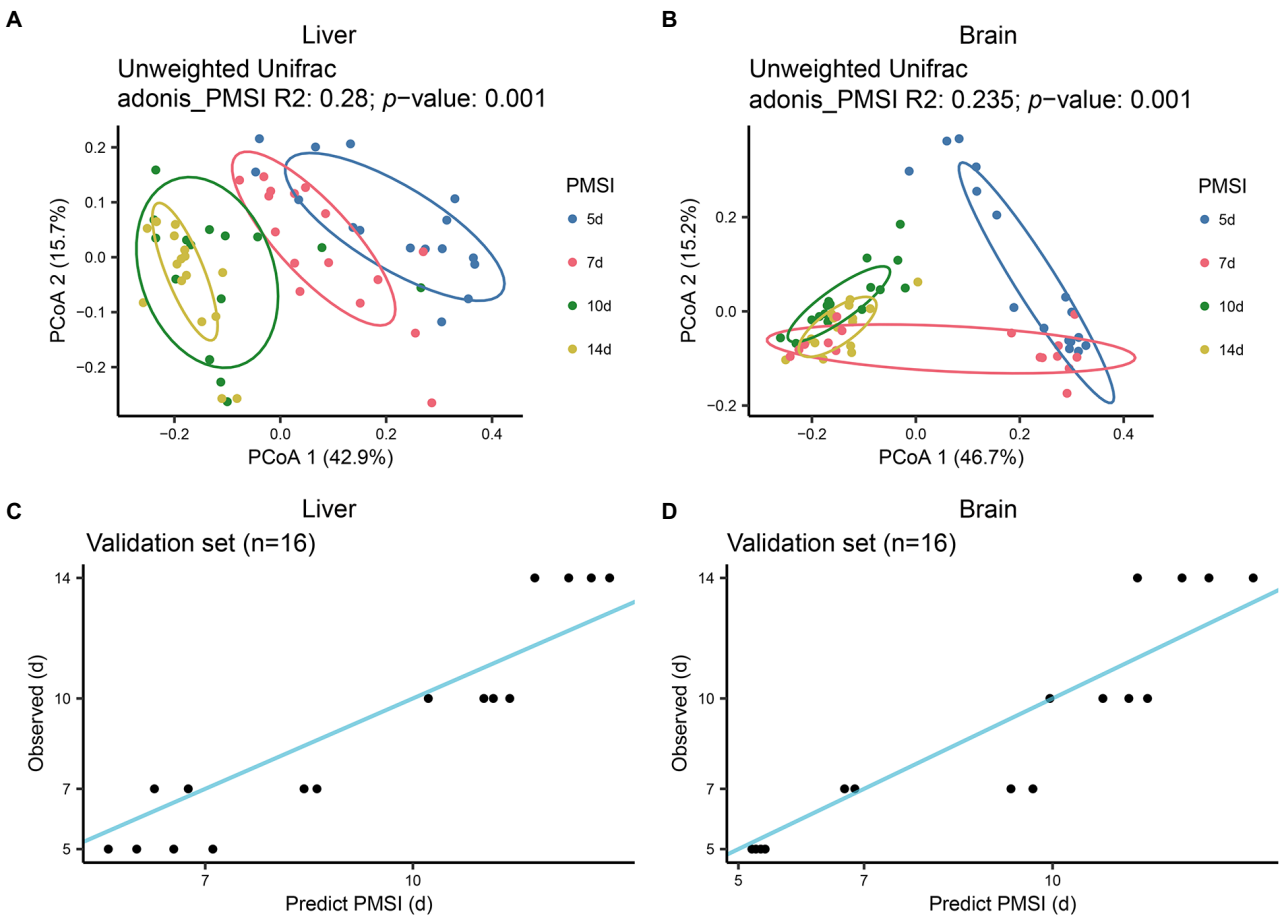
Successional dynamics of microbial communities in the liver and brain, and performances of regression models for PMSI estimation. PCoA of bacterial communities in liver **(A)**, and brain **(B)**. Different colors indicate different PMSIs. Predicted PMSI versus actual PMSI for liver **(C)**, and brain **(D)**, samples were plotted with a superimposed one-to-one reference line. Dots represent samples from the validation experiment (*n* = 4 per PMSI).

The postmortem successions of microbial communities in liver and brain were assessed for PMSI estimation. PCoA based on unweighted UniFrac distance (Figures 4A,B) revealed obvious chronological ordination along PCoA1 both in liver and brain. Samples were briefly clustered into two categories; 5–7 days and 10–14 days. Subsequently, the relative abundance of microbiota at the level of ASV was analyzed with the RF algorithm to establish PMSI estimation models (the initial models). The variance explained in the liver model was 81.46%, and the variance explained in the brain was 82.45%. The regression models obtained satisfactory performances from the experimental data (liver MAE 1.137 d ± 0.115 d; brain MAE 1.114 d ± 0.111 d). The validation data were used to verify the efficiency of the models, and the MAE ± SE values were 1.229 d ± 0.146 d for the liver and 1.077 d ± 0.231 d for the brain (Figures 4C,D; Table 2). These results suggested that microbiota in liver and brain could be used for estimating PMSI in late-phase submerged corpses.

**Table 2:** Prediction results of validation samples derived from the initial and refined regression models.

Sample	Group	Observed	Predic_	Error_	Predict_	Error_	Predict_	Error_	Predict_	Error_
			initial_	initial_	refined_	refined_	initial_	initial_	refined_	refined_
			liver*	liver*	liver*	liver*	brain*	brain*	brain*	brain*
V1	D	5	5.6	0.6	5.995	0.995	5.215	0.215	5.037	0.037
V2	D	5	6.01	1.01	6.246	1.246	5.357	0.357	5.216	0.216
V3	D	7	6.266	−0.734	6.257	−0.743	6.689	−0.311	6.737	−0.263
V4	D	7	6.755	−0.246	7.051	0.051	6.853	−0.147	6.997	−0.003
V5	D	10	10.223	0.223	10.09	0.09	10.8	0.8	10.873	0.873
V6	D	10	11.025	1.025	11.024	1.024	9.957	−0.043	9.876	−0.124
V7	D	14	12.251	−1.749	12.846	−1.154	12.488	−1.512	12.955	−1.045
V8	D	14	11.762	−2.238	11.433	−2.567	12.06	−1.94	12.545	−1.455
V9	PS	5	7.11	2.11	7.345	2.345	5.426	0.426	5.307	0.307
V10	PS	5	6.545	1.545	6.677	1.677	5.278	0.278	5.009	0.009
V11	PS	7	8.428	1.428	8.486	1.486	9.687	2.687	9.461	2.461
V12	PS	7	8.614	1.614	9.171	2.171	9.338	2.338	9.404	2.404
V13	PS	10	11.163	1.163	11.574	1.574	11.213	1.213	11.364	1.364
V14	PS	10	11.4	1.4	12.039	2.039	11.511	1.511	11.92	1.92
V15	PS	14	12.578	−1.422	13.463	−0.537	11.351	−2.649	11.327	−2.673
V16	PS	14	12.843	−1.157	13.187	−0.813	13.193	−0.807	13.336	−0.664

* Predict_initial and Error_initial: Prediction results of validation samples derived from the regression model based on all ASVs. Predict refined and Error refined: Prediction results of validation samples derived from the regression model established by selected taxa (liver 18 ASVs; brain 26 ASVs). PMSI was measured in units of days.

**Figure 5:**
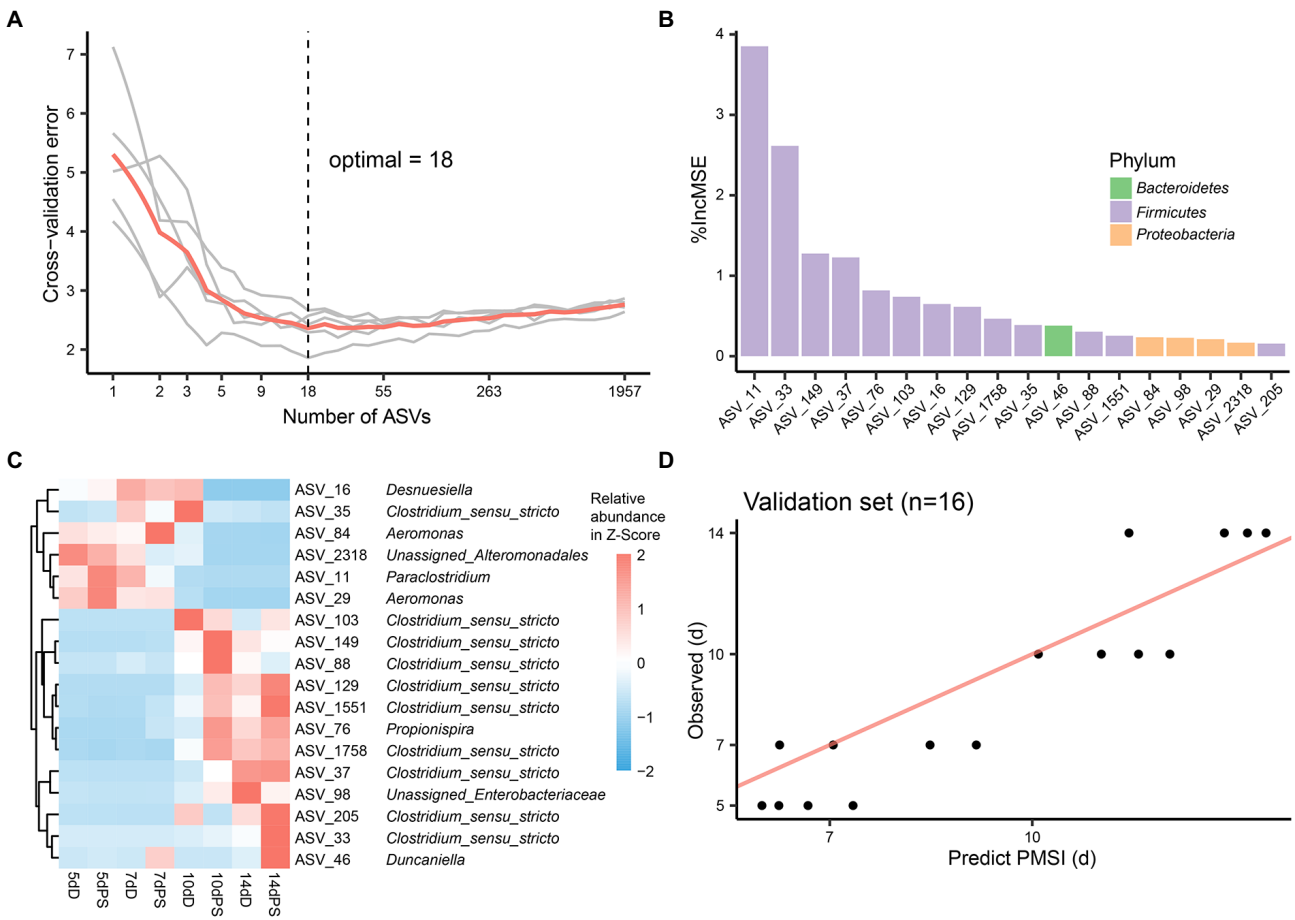
Liver biomarker identification and validation for PMSI estimation. **(A)** Cross-validation results of the initial model established using liver microbial communities. **(B)** The top 18 ASVs were identified by the RF algorithm. Biomarker taxa were ranked in decreasing order of importance (i.e., %IncMSE). **(C)** Heatmap demonstrating dynamic changes in abundance of the top 18 PMSI-predictive biomarkers. **(D)** Predicted PMSI versus actual PMSI for liver samples obtained by the refined regression model plotted with a superimposed one-to-one reference line. The dots represent liver samples from the validation experiment (*n* = 4 per PMSI).

**Figure 6:**
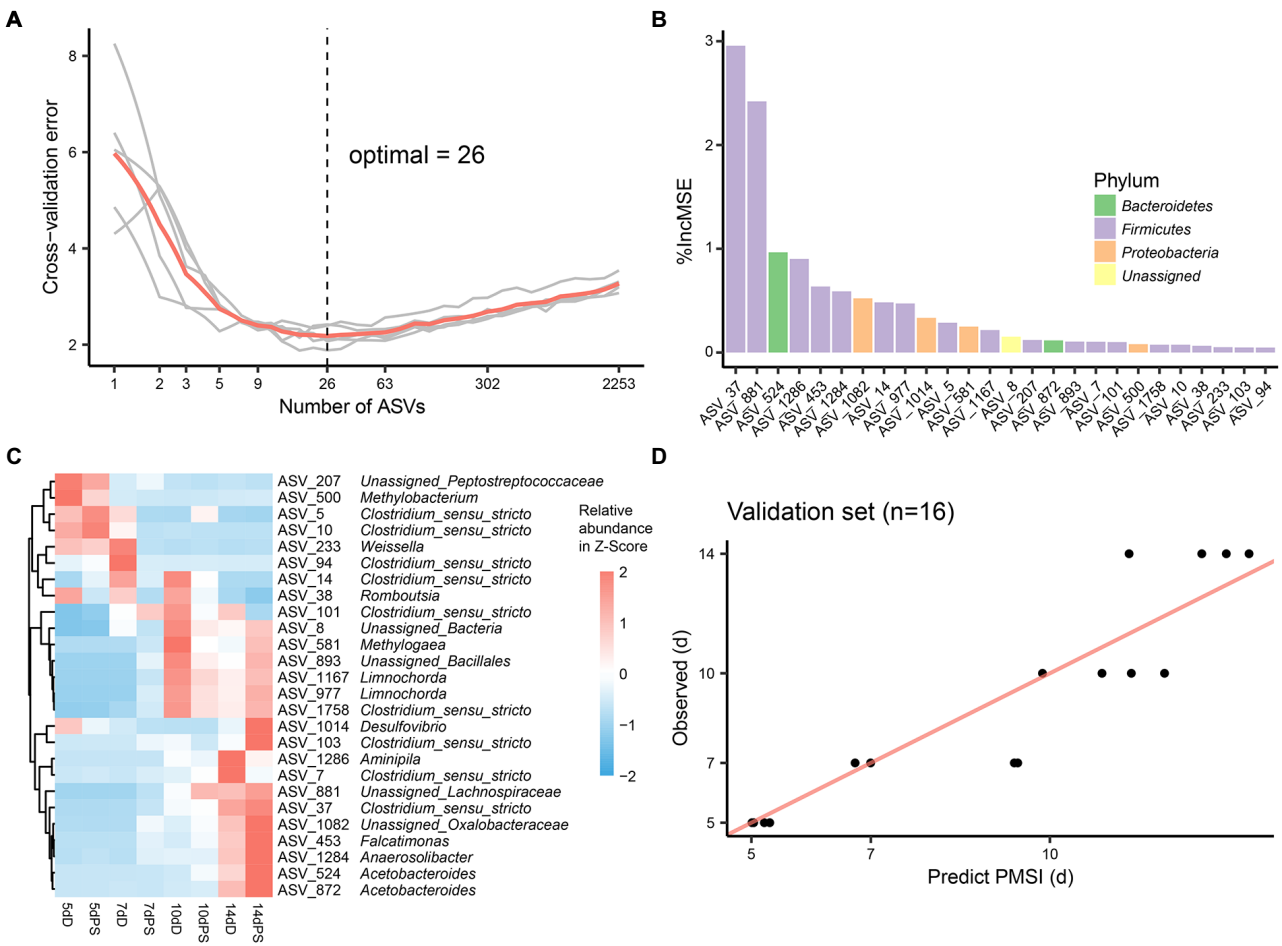
Brain biomarker identification and validation for PMSI estimation. **(A)** Cross-validation result of the initial model established using brain microbial communities. **(B)** The top 26 ASVs were identified by the RF algorithm. Biomarker taxa were ranked in decreasing order of importance (i.e., %IncMSE). **(C)** Heatmap demonstrating dynamic changes in abundance of the top 26 PMSI-predictive biomarkers. **(D)** Predicted PMSI versus actual PMSI for brain samples obtained by the refined regression model plotted with a superimposed one-to-one reference line. Dots represent brain samples from the validation experiment (*n* = 4 per PMSI).

Though these models demonstrated satisfactory accuracy for PMSI prediction, there may be some ASVs contributing less to the models. Hence, cross-validation was performed to select the top informative indicator set (Figures 5A, 6A). Eighteen ASVs with high PMSI-discriminatory importance were selected as potential biomarkers in the liver, and 26 were selected in the brain (Figures 5B,C, 6B,C). Some microbes decreased in relative abundance over the PMSI, while others increased. According to species annotation, *Firmicutes* (13 ASVs in liver and 19 in brain) were dominant. Significant taxa in the liver were related to *Paraclostridium*, *Clostridium_sensu_stricto*, *Propionispira*, *Desnuesiella*, *Duncaniella*, and *Aeromonas* at the genus level. The microbes in brain mainly belonged to *Clostridium_sensu_stricto*, *Acetobacteroides*, and *Limnochorda*. Though the compositions of these indicator sets differed in liver and brain, *Clostridium_sensu_stricto* accounted for the dominant microbiota (10 ASVs in the liver and 9 in the brain). Three biomarkers (ASV 37, ASV 103, and ASV 1758) were shared between the two organs, which were assigned to *Clostridium_sensu_stricto*. A similar pattern in relative abundance for ASV 37 and ASV 1758 was observed in liver and brain. Lastly, the relative abundance of microbiota from the most informative indicator sets was further regressed against PMSI (the refined models). Compared with the initial models, the explained variances in these refined models (liver 85.71%, brain 85.78%) were slightly increased. The MAEs of the refined models were similar to those of the initial models with respect to data from exploratory experiments (liver 0.906 d ± 0.114 d, brain 0.911 d ± 0.113 d) and validation experiments (liver 1.282 d ± 0.189 d, brain 0.989 d ± 0.237 d; Figures 5D, 6D; Table 2). These results indicated that these refined models based on selected microbial communities in liver and brain had powerful potential for late-phase PMSI estimation.

## Discussion

Many studies have investigated carrion microbial succession using high-throughput sequencing in terrestrial habitats (Metcalf et al., 2016, 2017; Liu et al., 2021), but there are comparatively few studies on microbial communities in the internal organs in aquatic ecosystems. Thus, in the current study, we characterized shifts in bacterial communities in the livers and brains of mouse cadavers in natural freshwater, and compared differences between drowned corpses and those only subjected to postmortem submersion. The temporal succession of microbiota colonization in some liver and brain samples from these corpses was informative for PMSI estimation. Bacterial communities in the liver and brain were of little use for drowning identification.

Many internal organs including liver and brain are believed to be sterile in healthy living hosts (Javan et al., 2016). After death, various bacteria begin to spread throughout the entire corpse. The microbes discovered in these organs are mainly associated with decomposition. In the present study, most of the samples collected on days 0 to 3 postmortem were not qualified for subsequent detection, implying that there was little to no bacteria proliferating in the viscera at these timepoints. In our previous study, liver samples collected at 3 days postmortem could meet the requirements of next-generation sequencing (Wang et al., 2020). Because temperature is one of the most important environmental factors affecting the succession of microbes (Zhou et al., 2021), a reasonable explanation for the discrepancy between the previous study and the current study is that the ambient temperature in the present study (5 to 10°C) was substantially lower than that in the previous study (15 to 25°C), resulting in limited growth of microorganisms. This result suggests that in follow-up translational studies, the concept of accumulated degree-days, which integrates postmortem interval and ambient temperature (Heaton et al., 2010), should be used to minimize error caused by temperature fluctuation. Additionally, a previous study reported that liver remains sterile up to 5 days after death (Tuomisto et al., 2013). Our experiment further supported this finding. So, it is a wise choice to focus on the microbial communities in other organs when it comes to fresh corpses.

In the present study bacterial communities in the liver and brain from corpses at advanced stages of decay could not be used for drowning determination. There are likely several reasons for this. First, bacteria from the external environment and gut would influence the microbial community in viscera. Sterile organs could be colonized by infiltrating bacteria, and tissues where there is a specific microbiota can be contaminated (Wójcik et al., 2021). The genus *Aeromonas* is ubiquitous in freshwater but absent in the healthy human body (Goncalves Pessoa et al., 2019), and it has been documented as a potential bacterial marker of freshwater drowning (Kakizaki et al., 2011; Huys et al., 2012; Uchiyama et al., 2012). In the current study, however, there was no significant difference in the relative abundance of *Aeromonas* in liver between drowning and postmortem submersion from 5-day postmortem, indicating the between-group differences caused by the exogenous species were relatively subtle at 5 days. Due to drastic changes in the environment, water-derived microorganisms that entered the internal organs during drowning might die gradually, resulting in decreased contribution of water-derived bacteria. Cartozzo et al. (Cartozzo et al., 2021b) reported that microorganisms inherent to the surrounding water environment contributed little to the dominating bone microbial communities with respect to relative abundance. That report is concordant with the present study. These results indicated that some changes in the microenvironment or in the microbial community during drowning became less pronounced as the PMSI extended, making a drowning diagnosis more difficult at advanced stages of decomposition. Additionally, in the current study classification models based on liver and brain microbiota collected at 5 days and 7 days performed well in exploratory experiments, implying their potential usefulness for drowning identification. The liver model exhibited poor performance in validation experiments, however (AUC 0.56), probably due to the large individual differences. This finding should be viewed with caution due to the small sample size in the present study, and should be validated in future larger studies.

Despite its unsatisfactory performance in drowning identification, microbial information derived from liver and brain exhibited good chronological regularity conducive to use for PMSI estimation. Both of the models yielded satisfactory accuracy in independent validation samples, suggesting the usefulness of liver and brain microbiota for late-PMSI estimation. In the present study, the minimum cross-validation errors were obtained when using 18 ASVs for the liver and 26 ASVs for the brain. A list of candidate taxa, identified *via* analysis of cross-validation, changed in abundance over time. Some of the microbial taxa have also been reported in postmortem microbiomes in terrestrial carcass decomposition studies. For example, many selected indicators were assigned to *Clostridium_sensu_stricto*, which was also the dominant bacteria in terrestrial carcasses at 5–14-day postmortem. *Clostridium* is a widely variable oxygen-tolerant anaerobic genus found in diverse environments such as soil, freshwater, and marine sediments (Bergogne-Berezin and Towner, 1996), and it has been regarded as a key contributor to the general decomposition process on land (Hyde et al., 2015). The translocation and proliferation of *Clostridium* in postmortem human internal organs has been reported in several studies (Tuomisto et al., 2013; Javan et al., 2016). Recent research has even defined a new scientific concept, the “postmortem *Clostridium* effect”—which refers to the ubiquitous *Clostridium* spp. present during human decomposition (Javan et al., 2017). The current study demonstrates that this phenomenon can also be observed in submerged corpses. Further, some taxa unique to aquatic systems were proposed as indicators of PMSI. For instance, taxa defined as *Aeromonas* which gradually decreased in the liver could be used for PMSI estimation. *Desulfovibrio* is commonly found in aquatic environments (Amrani et al., 2014). *Methylogaea* is isolated from the soil-water interface of rice paddy fields (Tarlera, 2016). A species of *Limnochorda* has been isolated from sediment from a brackish meromictic lake (Watanabe et al., 2015). *Acetobacteroides* is found in reed swamps. The abundances of ASVs related to *Methylogaea*, *Limnochorda*, and *Acetobacteroides* were higher at the advanced decomposition stage, implying that sediment-dwelling bacteria may play an important role during degradation. This warrants further research. Lastly, these bioindicators were useful for PMSI estimation with high predictive accuracy (liver MAE 1.282 d ± 0.189 d; brain MAE 0.989 d ± 0.237 d).

Although some of the findings in the present study are novel, it should be viewed as an initial investigation into microbial succession associated with decomposition in a natural freshwater environment. The study had some limitations. Bacterial is reportedly more resistant to harsh environmental conditions (i.e., chemical and physical agents) due to the wall of peptidoglycan matrix, which renders bacteria applicable to studies in corpses at advanced stages of decomposition (Tozzo et al., 2020). Further research should monitor the entire decomposition process (e.g., fresh to skeletonization). The environmental conditions surrounding the cadaver influence the bacterial communities present and the stages of decomposition, but the present study was conducted at a single location during a single season. For broader application of the findings in forensic science, it would be helpful to develop reliable and robust databases of microbiomes obtained in multiple aquatic environments and seasons.

The succession of postmortem microbiota that colonize internal organs (including the gut, brain, liver, spleen, and heart) has proven useful for PMI estimation in terrestrial environments (Can et al., 2014; Javan et al., 2016). The present study provides novel and informative context for better understanding the decomposition processes that submerged corpses undergo, which have important implications for forensic practice. It also sheds new light on PMSI estimation based on the succession of microbial populations in liver and brain specimens from corpses in water.

## Data availability statement

The datasets presented in this study can be found in online repositories. The names of the repository/repositories and accession number(s) can be found at: https://www.ncbi.nlm.nih.gov/, PRJNA883605.

## Ethics statement

The animal study was reviewed and approved by Animal Experiment Committee of China Medical University.

## Author contributions

DG and RZ conceived and designed the research. LW and FZ performed the lab experiments and wrote the main manuscript text. HY, ZW, JL, and JP performed the animal experiments. FZ, KZ, and WD performed the bioinformatic analysis. All authors contributed to the article and approved the submitted version.

## Funding

This work was supported by grant from the National Key Research and Development Program of China (grant number 2022YFC3302002), National Natural Science Foundation of China (grant numbers 82271926, 81971793, 81772023, 81801874), and Shenyang Science and Technology innovation support plan for young and middle-age talent (grant number RC200412).

## Conflict of interest

The authors declare that the research was conducted in the absence of any commercial or financial relationships that could be construed as a potential conflict of interest.

## Publisher’s note

All claims expressed in this article are solely those of the authors and do not necessarily represent those of their affiliated organizations, or those of the publisher, the editors and the reviewers. Any product that may be evaluated in this article, or claim that may be made by its manufacturer, is not guaranteed or endorsed by the publisher.

## Footnotes

^1^ http://www.r-project.org/

^2^ https://cran.r-project.org/package=vegan
