# Supplementary material for: Microbial communities in the liver and brain are informative for postmortem submersion interval estimation in the late phase of decomposition: A study in mouse cadavers recovered from freshwater
Source: Front Microbiol. 2022 Nov 15;13:1052808. doi: 10.3389/fmicb.2022.1052808 (PMC9705336; doi:10.3389/fmicb.2022.1052808)
Supplement: Supplementary file 1 [file Data_Sheet_1.docx]

Supplementary Material

# Supplementary Figures and Tables

## Supplementary Figures


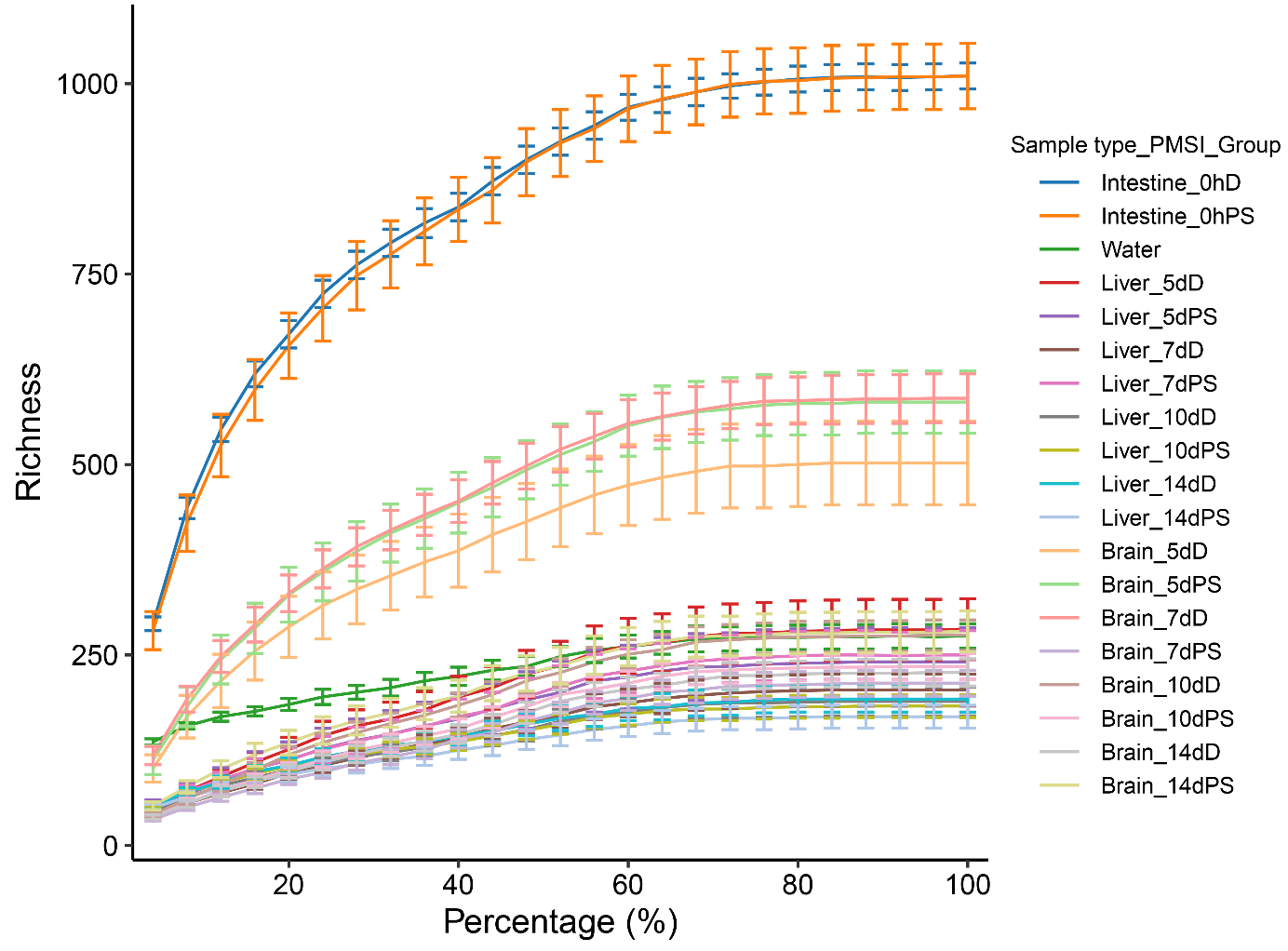


**Supplementary Figure 1.** Rarefaction curves of detected bacterial ASVs of the microbiota in different samples with increasing sequencing depth. Vertical bars represent the standard error.


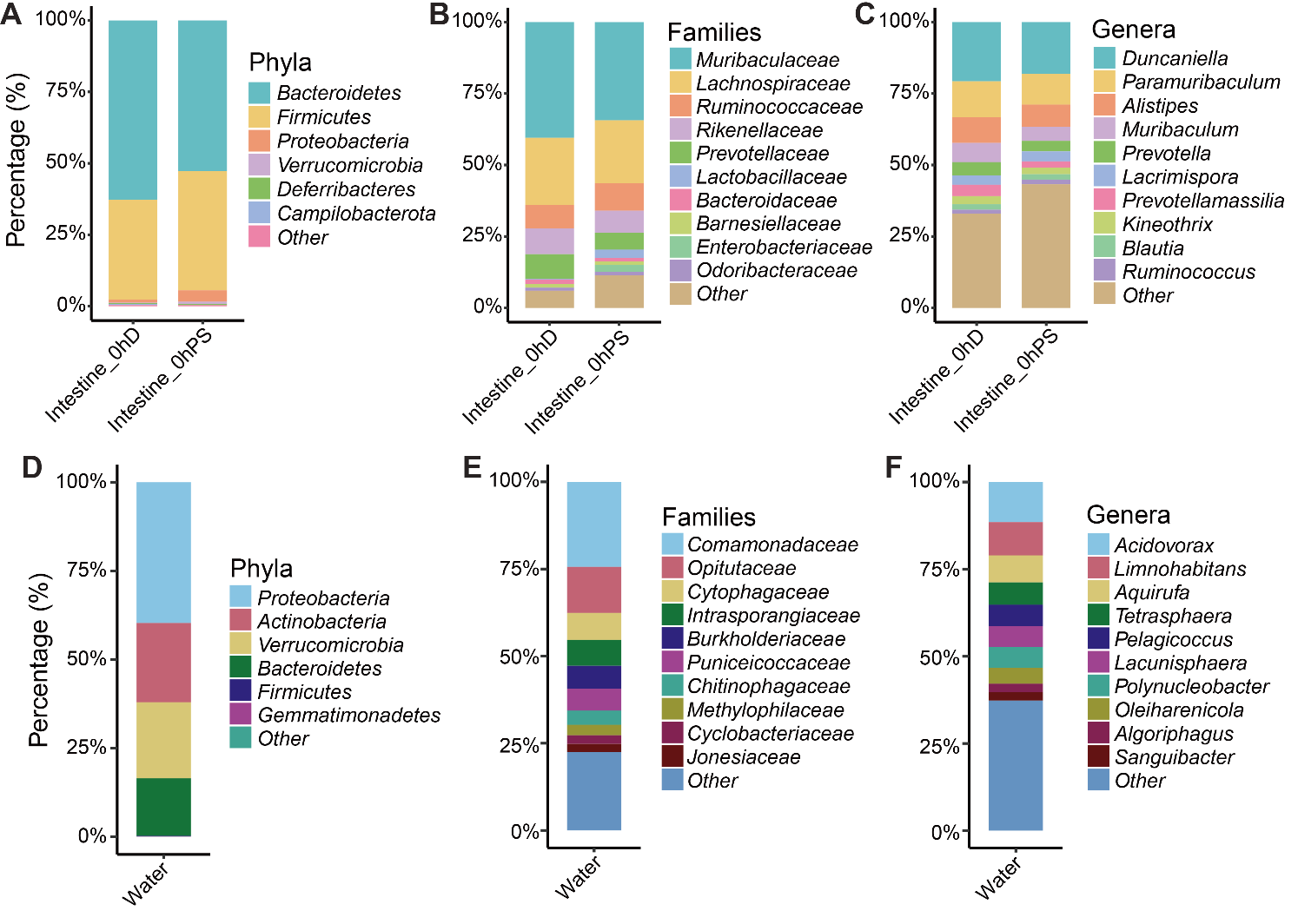


**Supplementary Figure 2.** Composition of microbial communities in gut and water. Stacked bar charts of the top 6 bacterial phyla (**A**), top 10 bacterial families (**B**), and top 10 bacterial genera (**C**) with the largest mean relative abundances in the intestine. Stacked bar charts of the top 6 bacterial phyla (**D**), top 10 bacterial families (**E**), and top 10 bacterial genera (**F**) with the largest mean relative abundances in water samples.

D, drowning group; PS, postmortem submersion group


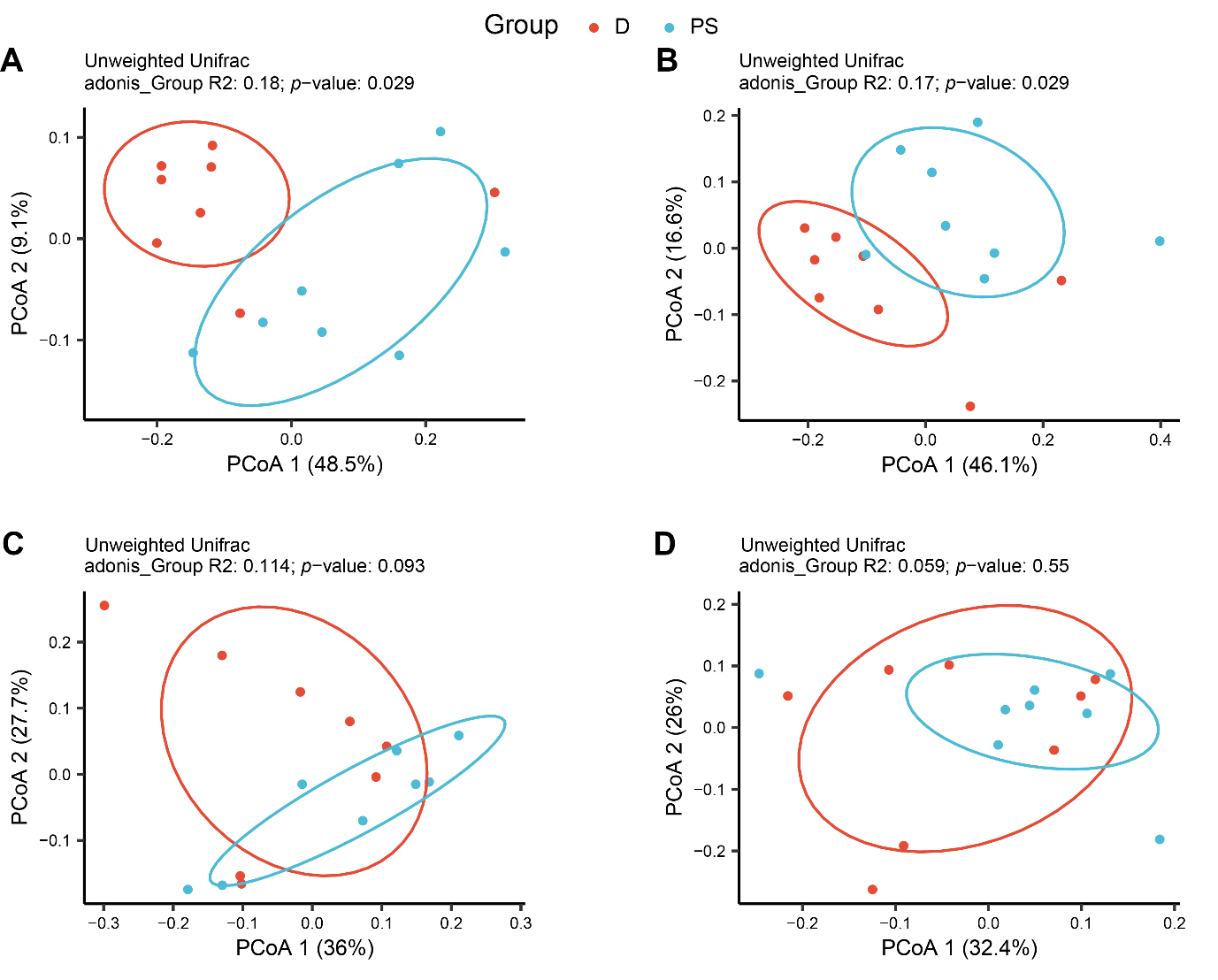


**Supplementary Figure 3.** Beta diversity analysis of microbiota in liver samples in drowning and postmortem submersion groups. (**A**) 5 d, (**B**) 7 d, (**C**) 10 d, (**D**) 14 d.

D, drowning group; PS, postmortem submersion group


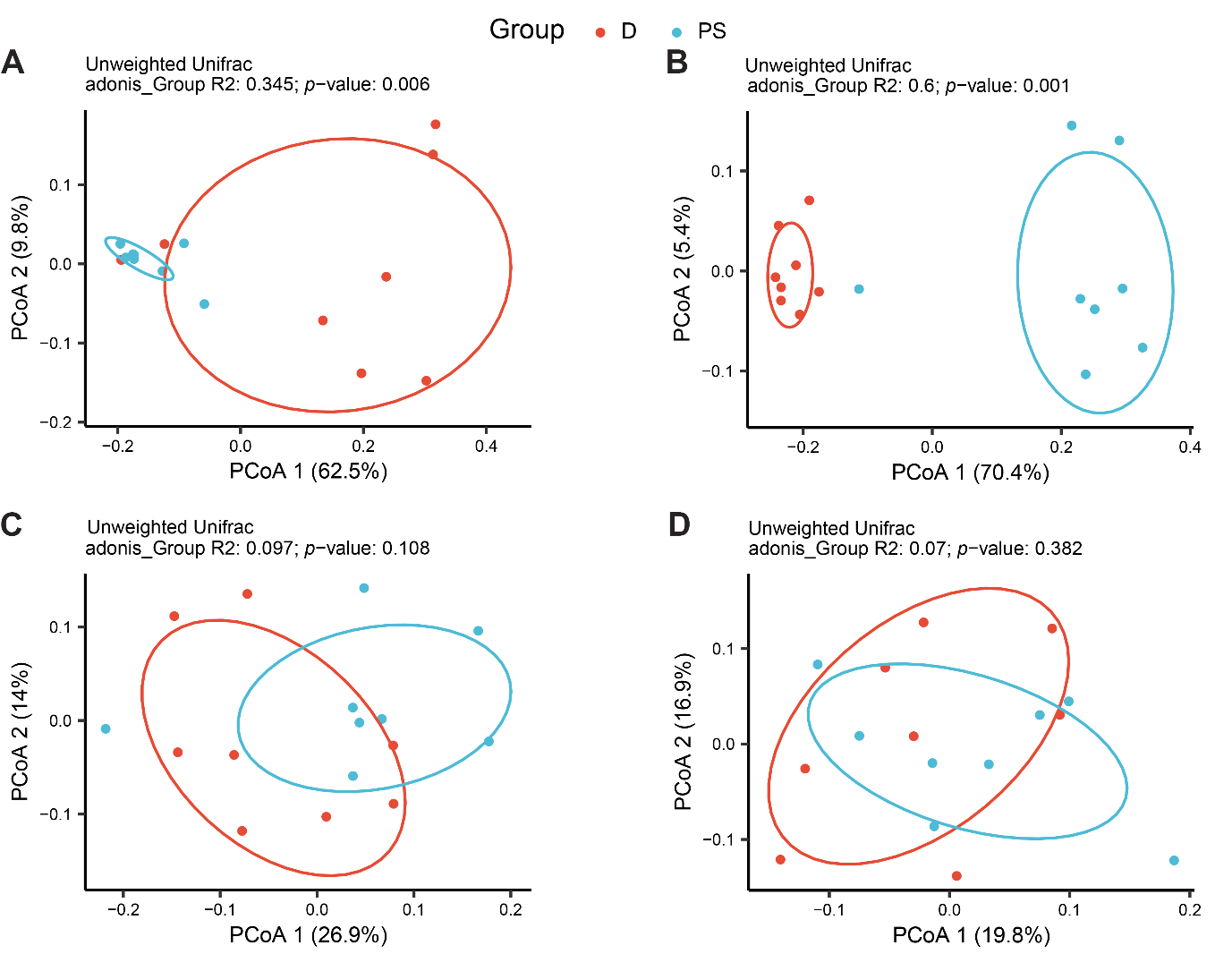


**Supplementary Figure 4.** Beta diversity analysis of microbiota in brain samples in drowning and postmortem submersion groups. (**A**) 5 d, (**B**) 7 d, (**C**) 10 d, (**D**) 14 d.

D, drowning group; PS, postmortem submersion group


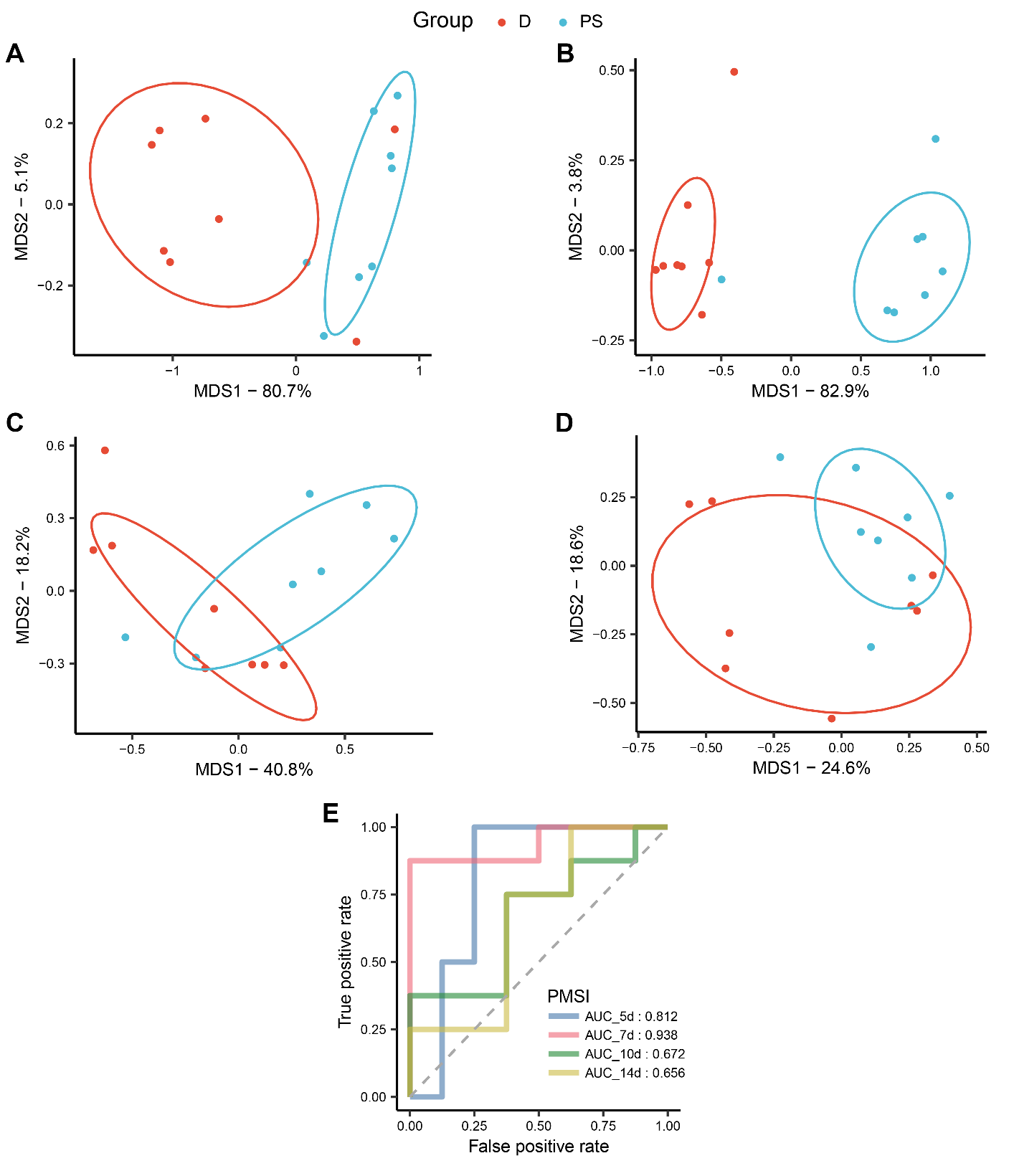


**Supplementary Figure 5.** Performance of RF classification model built on microbiota in the liver at each PMSI. (**A–D**) MDS plots generated by the learning algorithm RF comparing microbial communities in drowning and postmortem submersion groups. (**A**) 5 d, (**B**) 7 d, (**C**) 10 d, (**D**) 14 d. (**E**) ROC curves of the RF classification model on data from the exploratory experiment.

D, drowning group; PS, postmortem submersion group


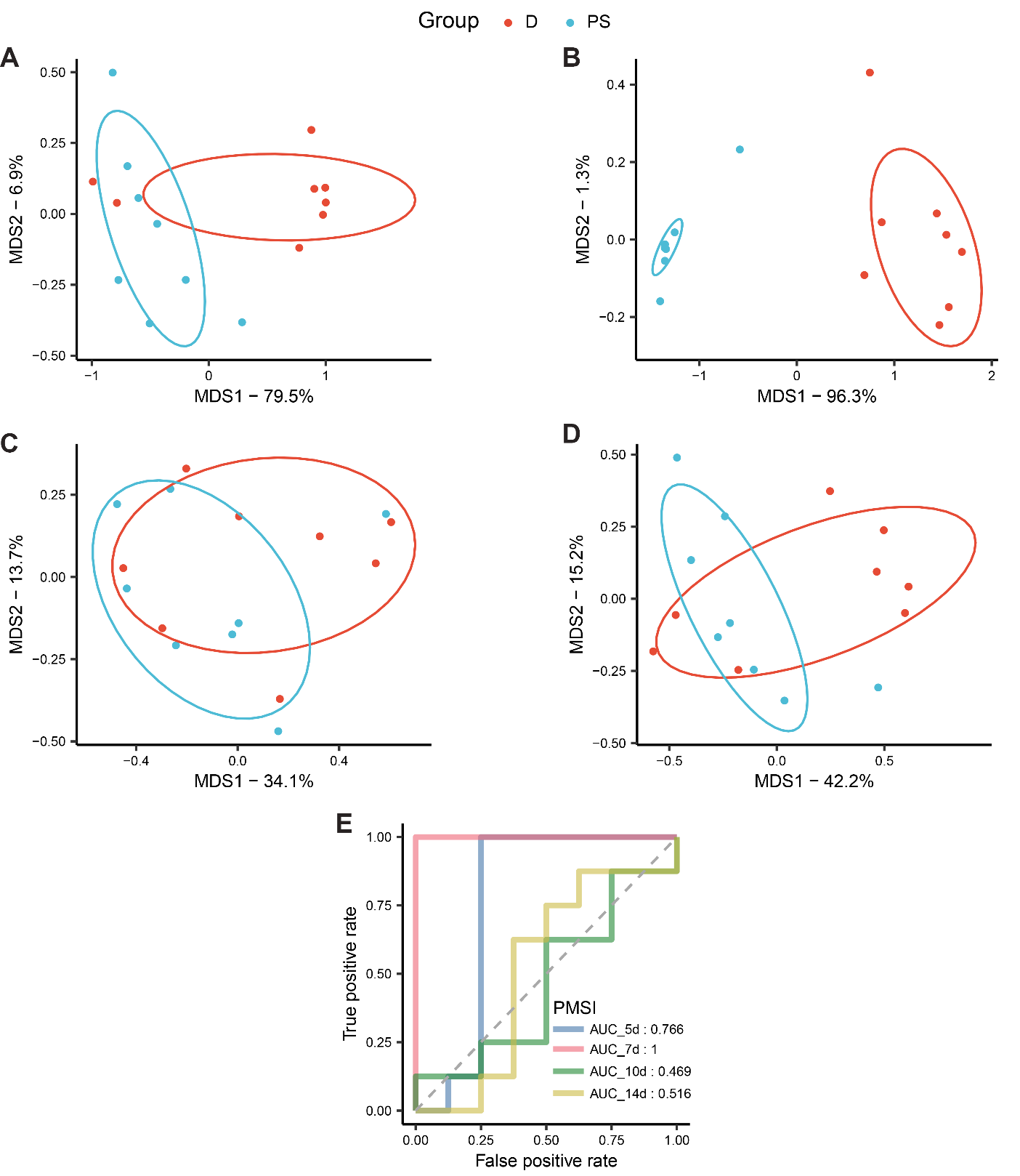


**Supplementary Figure 6.** Performance of RF classification model built on microbiota in the brain at each PMSI. (**A–D**) MDS plots generated by the learning algorithm RF comparing the microbial communities in drowning and postmortem submersion groups. (**A**) 5 d, (**B**) 7 d, (**C**) 10 d, (**D**) 14 d. (**E**) ROC curves of the RF classification model on data from the exploratory experiment.

D, drowning group; PS, postmortem submersion group


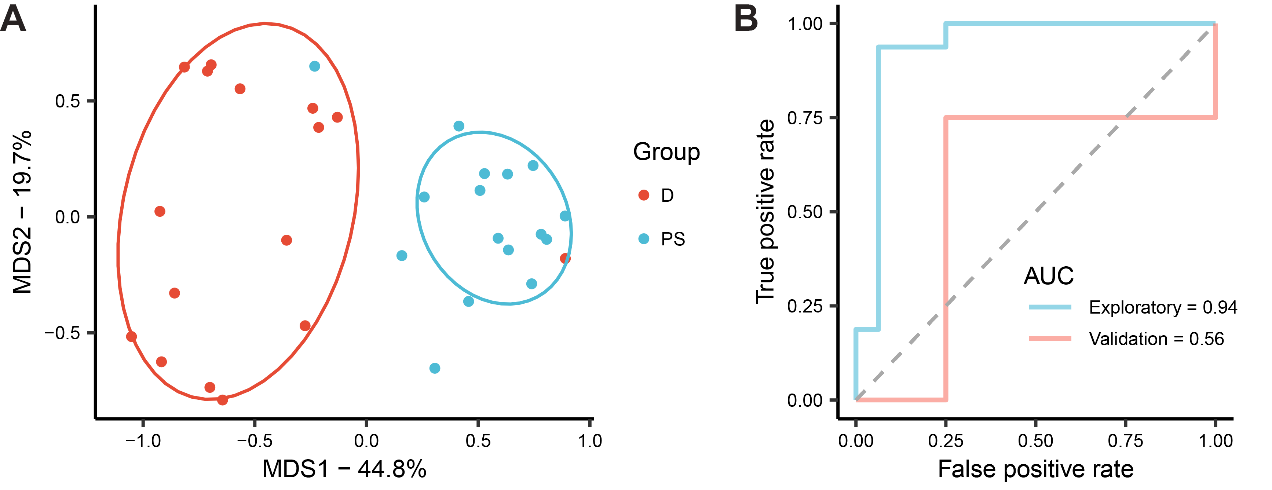


**Supplementary Figure 7.** Performance of RF classification model built on liver microbiota collected within 7 d postmortem. (**A**) MDS plot generated by the learning algorithm RF comparing the microbial communities in drowning and postmortem submersion groups. (**B**) ROC curves of the RF classification model on data from exploratory and validation experiments.

D, drowning group; PS, postmortem submersion group


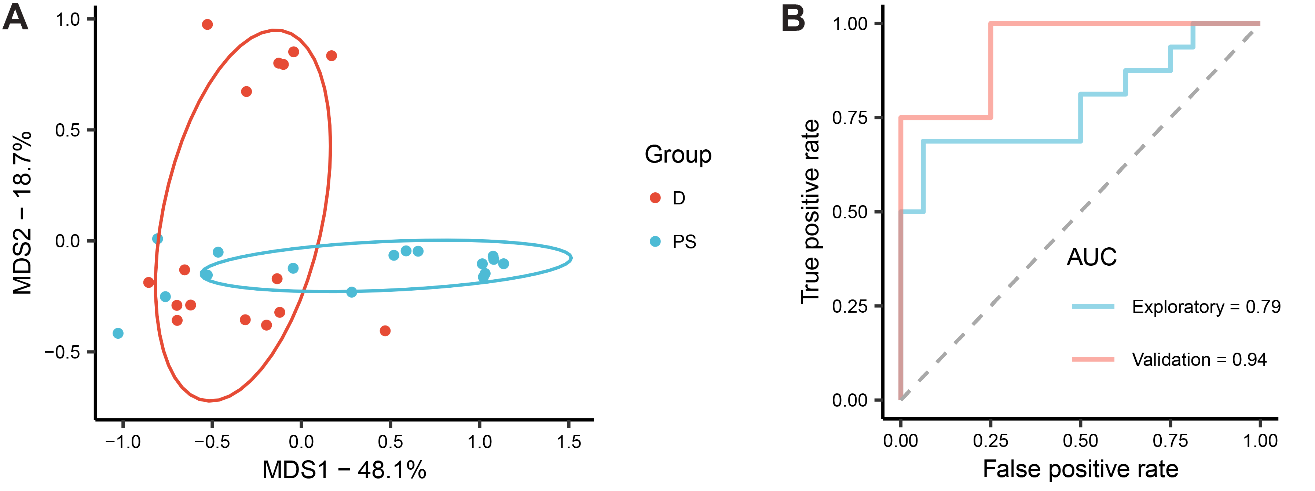


**Supplementary Figure 8.** Performance of RF classification model built on brain microbiota collected within 7 d postmortem. (**A**) MDS plot generated by the learning algorithm RF comparing the microbial communities in drowning and postmortem submersion groups. (**B**) ROC curves of the RF classification model on data from exploratory and validation experiments.

D, drowning group; PS, postmortem submersion group

## Supplementary Tables

**Supplementary Table 1** Experimental design and animal groups

| Postmortem submersion interval | Exploratory experiment (n) | | Validation experiment (n) | |
| --- | --- | --- | --- | --- |
|  | Drowning | Postmortem submersion | Drowning | Postmortem submersion |
| 0d | 8 | 8 | 2 | 2 |
| 0.25d | 8 | 8 | 2 | 2 |
| 0.5d | 8 | 8 | 2 | 2 |
| 1d | 8(*=1) | 8 | 2 | 2 |
| 3d | 8(*=1) | 8(*=2) | 2 | 2(*=1) |
| 5d | 8(*=8) | 8(*=8) | 2(*=2) | 2(*=2) |
| 7d | 8(*=8) | 8(*=8) | 2(*=2) | 2(*=2) |
| 10d | 8(*=8) | 8(*=8) | 2(*=2) | 2(*=2) |
| 14d | 8(*=8) | 8(*=8) | 2(*=2) | 2(*=2) |

* After quality control by agarose gel electrophoresis, samples met the requirements of microbiome profiling.
